# Root Exudates Shape Soil Organic Carbon Stabilization by Controlling Microbial Necromass Formation Under Long-Term Nitrogen Fertilization

**DOI:** 10.3390/microorganisms14061295

**Published:** 2026-06-08

**Authors:** Zheng Jiang, Cong Wang, Huifeng Sun, Jining Zhang, Xianxian Zhang, Sheng Zhou

**Affiliations:** 1 Eco-Environmental Protection Research Institute, Shanghai Academy of Agricultural Sciences, Shanghai 201403, China; jiangzheng@saas.sh.cn (Z.J.); wangcong@saas.sh.cn (C.W.); hfsun2010@126.com (H.S.); j.n.zhang@163.com (J.Z.); xixizi01090@163.com (X.Z.); 2 Shanghai Engineering Research Center of Low-carbon Agriculture (SERCLA), Shanghai 201415, China; 3 Key Laboratory of Low-Carbon Green Agriculture in Southeastern China, Ministry of Agriculture and Rural Affairs, Shanghai 201403, China

**Keywords:** nitrogen fertilization, root exudates, soil organic carbon, microbial necromass

## Abstract

Soil organic carbon (SOC) stabilization is largely governed by the accumulation of microbial necromass carbon, yet how stabilization is influenced by root exudation remains poorly understood, particularly under long-term nitrogen (N) fertilization. Here, we investigated SOC dynamics in paddy soil under long-term N fertilization (12 years, urea) at four application rates (0, 100, 200, and 300 kg N ha^−1^). We quantified root exudate inputs, microbial community composition, microbial necromass, and soil physicochemical properties to identify the primary drivers of SOC accumulation. Our results showed that SOC content increased progressively with N application rate and these changes were attributable to N-induced increases in root exudate inputs, alterations to microbial community structure, and enhanced accumulation of microbial necromass. Structural equation modeling further revealed that root exudates served as a critical linkage between N fertilization, microbial necromass accrual, and SOC stabilization. Taken together, our findings imply that long-term N fertilization enhances SOC accumulation by stimulating root exudate-driven microbial necromass accrual, highlighting the pivotal role of plant–soil interactions in regulating carbon dynamics in rice agroecosystems.

## 1. Introduction

Soil organic carbon (SOC) represents the largest terrestrial carbon (C) reservoir, playing a central role in climate regulation, soil fertility, and ecosystem productivity [1,2]. Nitrogen (N) availability is a key constraint on plant productivity, yet its effects on SOC storage and turnover remain highly context-dependent and a major source of uncertainty in C-climate feedback predictions [3,4]. Moderate N inputs can stimulate plant biomass and belowground C inputs via roots and rhizodeposition, promoting SOC accumulation in N-limited systems [5,6]. However, enhanced microbial activity may simultaneously accelerate native organic matter decomposition through rhizosphere priming, partially offsetting these gains [7,8]. Conversely, sustained or excessive N enrichment can destabilize SOC pools by restructuring microbial communities, reducing fungal dominance, altering extracellular enzyme activities, and decreasing microbial C use efficiency, ultimately accelerating SOC turnover and weakening long-term C sequestration [9,10]. Understanding the plant–soil–microbe interactions governing belowground C inputs and microbial C transformation is therefore essential for elucidating how long-term N fertilization regulates SOC stabilization and for developing evidence-based N management strategies [11,12].

Root exudates represent a major pathway of belowground C input, providing readily available substrates that sustain microbial growth and activity in the rhizosphere while shaping microbial community composition and functional traits [13,14]. These labile C inputs can strongly influence SOC dynamics by regulating microbial C use, rhizosphere priming, and the formation of microbial-derived organic matter [7,8]. Long-term N fertilization can modify root-derived C inputs by altering plant nutrient status and stoichiometric demand, particularly by intensifying relative phosphorus (P) limitation under elevated N availability [15,16]. Under such conditions, plants may increase the release of organic acids, phosphatases, and other rhizosphere compounds to mobilize soil P, thereby changing rhizosphere C fluxes and microbial nutrient-acquisition strategies [17,18]. However, how long-term N fertilization regulates C inputs from root exudation, and the extent to which these changes drive microbial community dynamics and SOC stabilization, remain poorly resolved.

The accumulation of microbial necromass is increasingly recognized as the primary pathway through which exudate-driven microbial processes contribute to SOC accumulation [11,19]. Its formation is co-governed by microbial growth, biomass turnover, and community composition [20,21]. The microbial C pump framework posits that labile organic inputs from plants contribute to stable SOC pools by promoting microbial anabolism and iterative necromass accumulation [22,23]. Root exudate inputs selectively stimulate the proliferation of distinct microbial guilds, thereby altering necromass production rates and community-level necromass composition [24,25]. Previous studies have confirmed that rhizodeposition (i.e., root exudation of organic compounds into soil) promotes the formation of necromass through microbial assimilation pathways, thereby strengthening the microbial C pump [5,26]. However, labile root-derived C also triggers priming effects that accelerate the decomposition of native SOC and the mineralization of necromass C, counteracting long-term necromass accumulation [7,8,27]. The balance between necromass formation and decomposition under variable N regimes thus constitutes a central yet poorly quantified control on SOC stabilization [28,29]. Systematic investigation of the linkages among root exudate C inputs, microbial community dynamics, necromass accumulation, and SOC stabilization under different levels of N application may therefore help elucidate how N nutrient influences SOC accumulation [25].

Paddy fields support food security for more than half of the world’s population while simultaneously functioning as significant soil C reservoirs and greenhouse gas sources [30,31]. The periodic flooding characteristic of paddy systems generates alternating anaerobic–aerobic conditions that regulate microbial metabolism, redox dynamics, and mineral-associated SOC stabilization [20,32]. Yet how N fertilization modulates root exudate C inputs to drive shifts in microbial community composition, necromass accumulation, and SOC stabilization remains a pivotal unresolved question for understanding the C-sink capacity of paddy ecosystems and guiding sustainable N management [7,8,29]. To address this gap, we conducted a 12-year field experiment across an N fertilization gradient (0, 100, 200, and 300 kg N ha^−1^), integrating in situ root exudate collection, amino sugar biomarker quantification, and microbial community structure analysis. We aimed to elucidate how N-induced changes in root exudate C inputs regulate microbial community dynamics, necromass accumulation, and SOC stabilization, and to evaluate the effects of different N input levels on soil C sequestration in paddy systems. We hypothesized that long-term N fertilization would increase root exudate C inputs, thereby restructuring microbial community composition, governing necromass production, and ultimately modulating SOC stabilization.

## 2. Materials and Methods

### 2.1. Site Description and Experimental Design

The study was conducted at the Zhuanghang Experimental Station, Fengxian District, Shanghai, China (30°53′ N, 121°23′ E), operated by the Shanghai Academy of Agricultural Sciences. Mean annual precipitation at the site is 1383 mm, and mean annual air temperature is 17.0 °C. Soil in the area is classified as a typical Haplaquept (USDA Soil Taxonomy), with 47.2% silt, 49.9% sand, and 2.8% clay in the 0–20 cm layer. At the beginning of the experiment, soil pH was 7.6, total nitrogen was 1.4 g kg^−1^, and SOC was 13.7 g kg^−1^. The station operates under a long-term rice-wheat rotation system [33].

The long-term fertilization experiment was initiated during the 2012 rice-growing season, with treatments consistent with local agronomic practices and previous field trials [34]. Twelve plots (each 7 × 8 m) were established, comprising four N fertilization treatments in triplicate: N0 (no N input), N100 (100 kg N ha^−1^), N200 (200 kg N ha^−1^), and N300 (300 kg N ha^−1^) in rice season. N was applied as urea at a ratio of 5:3:2 ratio (basal: tillering: heading stage). All experimental plots received uniform phosphorus (P) and potassium (K) applications. Phosphorus was applied as a basal dressing at 100 kg P_2_O_5_ ha^−1^, while potassium chloride was applied at 225 kg K_2_O ha^−1^, with 44% incorporated as a basal application and the remaining 56% top-dressed at the heading stage in rice season [33,35].

### 2.2. Root Exudate Carbon Flux

Root exudates were sampled at the tillering, booting, and filling stages of the 2023 growing season following the in situ method described by previous studies [36,37]. At each growth stage, root exudates were collected from three replicate plots per treatment (*n* = 3 per treatment, 12 samples in total per stage). From each plot, one representative uniformly sized and healthy plant was selected from the central area to avoid edge effects. In brief, the selected plant was gently rinsed to remove adhering soil, and dead root segments were removed using stainless steel forceps. The plant was then submerged in a conical flask containing 100 mL of a 1:10 (*v*/*v*) mixture of local soil and deionized water for 24 h under ambient field conditions. Subsequently, roots were transferred into fresh flasks containing 100 mL of nutrient solution (0.3 mM CaCl_2_·2H_2_O, 0.2 mM MgSO_4_·7H_2_O, 0.2 mM K_2_SO_4_, and 0.1 mM KH_2_PO_4_) and shaken at 60 rpm for 2 h at 25 °C. The collected solution was passed through a 0.22 μm membrane filter and stored at −20 °C prior to dissolved organic C quantification using a TOC analyzer (TOC-L CPH, Shimadzu, Kyoto, Japan). Simultaneously, root systems from each plot were harvested, heat-killed at 105 °C for 30 min, and oven-dried to a constant mass at 60 °C to obtain fine root biomass. The mass-specific exudation rate (μg C g^−1^ root dry mass h^−1^) was calculated by dividing the total dissolved organic C recovered from the 2 h incubation by the oven-dried fine root. Field observations confirmed a consistent growing season length of 120 days. Seasonal root exudate C flux (g C m^−2^ a^−1)^ was calculated by determining the instantaneous C flux at each of three phenological stages (tillering, booting, and filling) as the product of the exudation rate and fine root biomass. The three instantaneous fluxes were averaged, and the resulting mean daily flux was multiplied by the 120-day growing season to obtain the total seasonal flux.

### 2.3. Soil Sampling and Measurement

Soil samples were collected from the topsoil layer (0–20 cm) of each plot in October 2023 after rice harvest. Four N fertilization treatments (N0, N100, N200, and N300) were applied, each with three replicate plots (*n* = 3 per treatment, 12 samples in total). Within each plot, twelve soil cores were randomly collected and thoroughly mixed to obtain one composite sample. The samples were then passed through a 2 mm sieve to remove stones and root debris and air-dried for subsequent analyses. Soil organic carbon (SOC) and total nitrogen (TN) concentrations were measured using an elemental analyzer (Vario EL Cube, Langenselbold, Germany) after carbonate removal by HCl treatment. Total phosphorus (TP) was determined by inductively coupled plasma–optical emission spectrometry (ICP-OES; Thermo Fisher Scientific iCAP 7000, Braunschweig, Germany). Dissolved inorganic nitrogen (DIN) was analyzed using a flow injection analyzer (AutoAnalyser III, SEAL Analytical, Norderstedt, Germany). Available phosphorus (AP) was extracted with 0.5 M NaHCO_3_ and quantified using the molybdenum blue colorimetric method at 700 nm. Microbial biomass carbon (MBC) and nitrogen (MBN) were measured by chloroform fumigation–extraction, followed by analysis using a total organic C analyzer. Soil pH was determined in a 1:2.5 soil-to-water suspension.

### 2.4. Soil Phospholipid Fatty Acids

Phospholipid fatty acids were extracted from three independent soil samples per treatment (*n* = 3 per treatment, 12 samples in total). For each sample, 3.0 g of freeze-dried soil was used for PLFAs extraction. Lipids were extracted and separated into fractions, followed by quantification using gas chromatography equipped with an Agilent 19091B-102 capillary column (30 m × 0.32 mm × 0.25 μm) [38]. PLFA profiles were identified using the Sherlock Microbial Identification System. Gram-positive bacteria (GP) were represented by i14:0, i15:0, a15:0, i16:0, i17:0, and a17:0, whereas Gram-negative bacteria (GN) were characterized by 16:1ω7c, 18:1ω7c, cy17:0, and cy19:0. Total bacterial biomass was estimated as the combined abundance of GP and GN markers. Arbuscular mycorrhizal fungi (AMF) were indicated by 16:1ω5c, while actinomycetes (ACT) were evaluated based on 10Me-16:0, 10Me-17:0, and 10Me-18:0. General fungal biomass was represented by 18:1ω9 and 18:2ω6,9. Total PLFA content was calculated as the sum of individual fatty acid profiles. The fungal-to-bacterial ratio was obtained by dividing fungal PLFAs by bacterial PLFAs.

### 2.5. Soil Amino Sugars

Amino sugars were analyzed from three independent freeze-dried soil samples per treatment (*n* = 3 per treatment, 12 samples in total). Each sample was subjected to acid hydrolysis using freeze-dried soil containing 0.3 mg N [39]. The hydrolysates were processed with appropriate internal and recovery standards, and the target amino sugars, including mannosamine (ManN), muramic acid (MurA), glucosamine (GluN), and galactosamine (GalN), were separated using an HP-5 capillary column and quantified by gas chromatography. Microbial necromass carbon (MNC) was estimated according to a previous study [21], with GluN and MurA used as microbial necromass biomarkers. Bacterial necromass carbon (BNC), fungal necromass carbon (FNC) and total microbial necromass carbon (MNC) were calculated as follows:

FNC = (GluN/179.2 − 2 × MurA/251.2) × 179.2 × 9(1)

BNC = MurA × 45(2)

MNC = FNC + BNC(3)

### 2.6. Statistical Analysis

Differences in root exudate C inputs, microbial communities, microbial necromass, and soil properties across N treatments were assessed using one-way ANOVA, after confirming normality (Shapiro–Wilk test) and homogeneity of variances (Levene’s test) (*p* > 0.05). Following a significant ANOVA result (*p* < 0.05), Tukey’s honest significant difference (HSD) post hoc test was applied for all pairwise comparisons among the four N levels (N0, N100, N200, N300). We also calculated Pearson correlation coefficients among variables describing SOC properties, microbial community characteristics, and microbial necromass. Structural equation modeling was performed using the “piecewiseSEM” (version 2.3.0) package in R to quantify the direct and indirect effects of soil properties and microbial characteristics on SOC content [40]. Considering the relationship between soil properties (TN, TP, DIN, AP, pH), microbial characteristics (MBC, MBN, PLFAs, GP, GN, Fungi, ACT, AMF, F:B), and microbial necromass (MNC, BNC, FNC), the first principal component derived from principal component analysis was used to replace the independent variables using the “FactoMineR” (version 2.8) package. Model fit was evaluated using Fisher’s C-statistic to test conditional independence, the associated *p*-value to assess overall goodness of fit (*p* > 0.05), and the Akaike information criterion (AIC) for model comparison (lower values indicate better fit). We used standardized path coefficients to quantify the strength and direction of relationships, with statistical significance assessed at *p* < 0.05. Coefficients of determination (R^2^) were used to assess the explained variance for each response variable [41]. All statistical analyses were conducted in R (version 4.3.1).

## 3. Results

**Figure 1:**
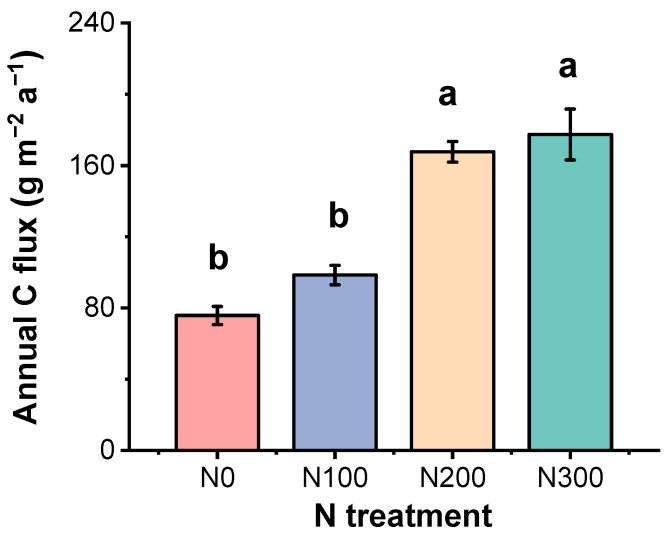
Effects of nitrogen (N) application rates on annual root exudate carbon (C) flux in paddy soil. N0, N100, N200, and N300 represent N application rates of 0, 100, 200, and 300 kg N ha^−1^, respectively. Error bars indicate standard error of the mean (*n* = 3), and different lowercase letters denote significant differences among treatments (*p* < 0.05).

As the rice growing season progressed, the rate of root exudate C inputs gradually declined, whereas root biomass continuously increased (Table S1). Nitrogen (N) fertilization significantly stimulated both root exudation and root biomass throughout the 120-day rice growing season, resulting in a marked increase in annual root-derived C flux with increasing N application rate. Compared with N0 (75.8 g m^−2^ a^−1^), annual C flux increased by 121.3% and 134.2% under N200 and N300, respectively, whereas no significant difference was observed between N100 and N0 (Figure 1).

**Figure 2:**
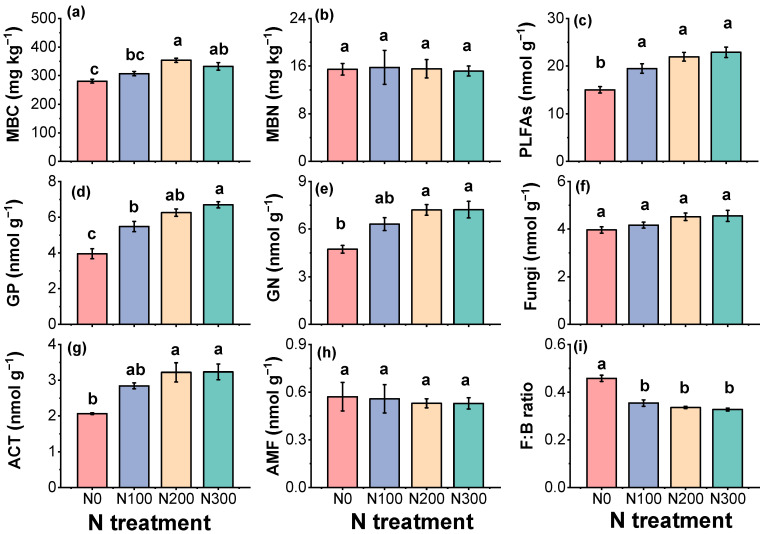
Effects of N application rates on soil microbial properties: (**a**) microbial biomass carbon (MBC), (**b**) microbial biomass nitrogen (MBN), (**c**) total phospholipid fatty acids (PLFAs), (**d**) Gram-positive bacteria (GP), (**e**) Gram-negative bacteria (GN), (**f**) fungi, (**g**) actinomycetes (ACT), (**h**) arbuscular mycorrhizal fungi (AMF), and (**i**) the fungal-to-bacterial ratio (F:B ratio). N0, N100, N200, and N300 represent N application rates of 0, 100, 200, and 300 kg N ha^−1^, respectively. Error bars indicate standard error of the mean (*n* = 3), and different lowercase letters denote significant differences among treatments (*p* < 0.05).

**Figure 3:**
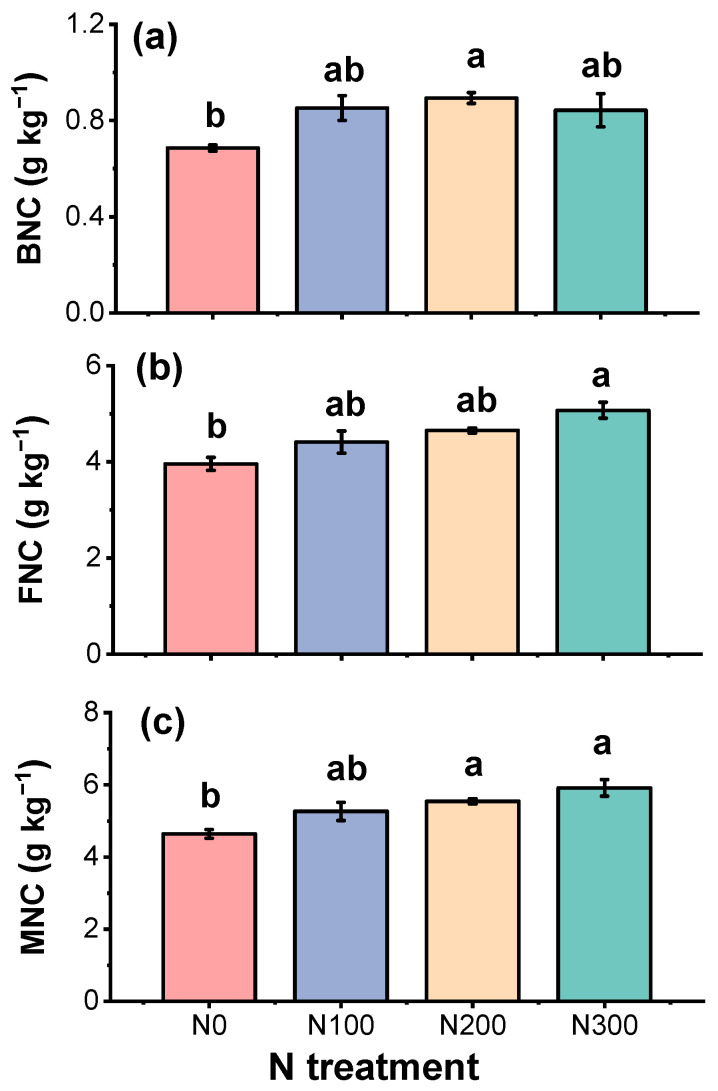
Effects of N application rates on soil microbial necromass carbon: (**a**) bacterial necromass carbon (BNC), (**b**) fungal necromass carbon (FNC), and (**c**) total microbial necromass carbon (MNC). N0, N100, N200, and N300 represent N application rates of 0, 100, 200, and 300 kg N ha^−1^, respectively. Error bars indicate standard error of the mean (*n* = 3), and different lowercase letters denote significant differences among treatments (*p* < 0.05).

N application induced pronounced changes in soil microbial biomass and community structure. Compared with N0, MBC increased significantly by 26.0% and 18.4% under N200 and N300, respectively. Total phospholipid fatty acids (PLFAs) increased progressively with increasing N application rate and reached a maximum under N300 (25.5 nmol g^−1^). Similar responses were observed for major microbial groups, including Gram-positive bacteria (GP), Gram-negative bacteria (GN), actinomycetes (ACT), and fungi, which reached 6.7, 7.2, 3.2, and 4.6 nmol g^−1^, respectively, under N300. In contrast, the fungal-to-bacterial ratio (F:B) decreased with increasing N application, reaching its lowest values under N300 treatment (0.33; Figure 2i). Nitrogen fertilization also promoted the accumulation of MNC. Compared with N0 (0.69 g kg^−1^), BNC increased by 30.4% under N200 treatment. FNC and total MNC also increased with increasing N application rate, reaching their highest values under N300 at 5.1 and 5.9 g kg^−1^, respectively (Figure 3).

**Figure 4:**
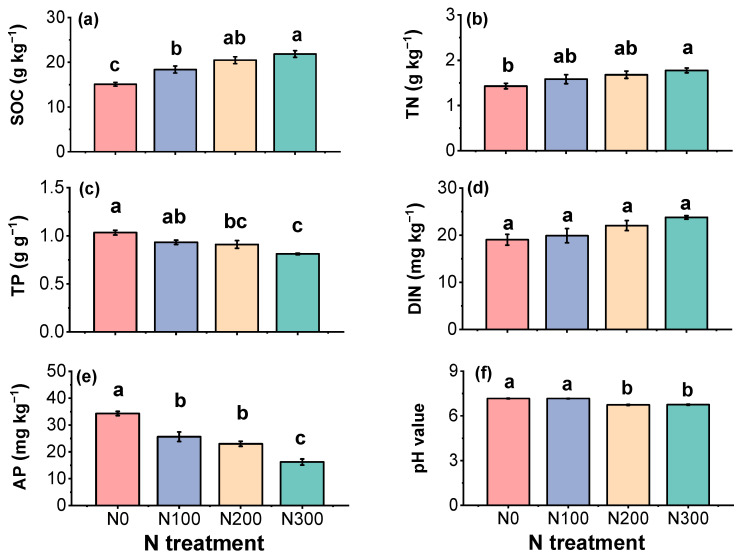
Effects of N application rates on soil physicochemical properties: (**a**) soil organic carbon (SOC), (**b**) total nitrogen (TN), (**c**) total phosphorus (TP), (**d**) dissolved inorganic nitrogen (DIN), (**e**) available phosphorus (AP), and (**f**) soil pH value. N0, N100, N200, and N300 represent N application rates of 0, 100, 200, and 300 kg N ha^−1^, respectively. Error bars indicate standard error of the mean (*n* = 3), and different lowercase letters denote significant differences among treatments (*p* < 0.05).

SOC increased progressively with N application and peaked under N300 at 21.9 g kg^−1^. Compared with N0, SOC was 35.6% and 44.8% higher under N200 and N300, respectively (Figure 4). Soil total nitrogen (TN) increased with increasing N application rate, reaching 1.8 g kg^−1^ under N300, corresponding to increases of 24.2% relative to N0 treatment. Conversely, total phosphorus (TP), available phosphorus (AP), and soil pH declined with increasing N application. Under N300, TP, AP, and pH decreased to 0.81 g kg^−1^, 16.2 mg kg^−1^, and 6.8, respectively, representing reductions of 21.9%, 52.8%, and 5.8% compared with N0 (Figure 4).

**Figure 5:**
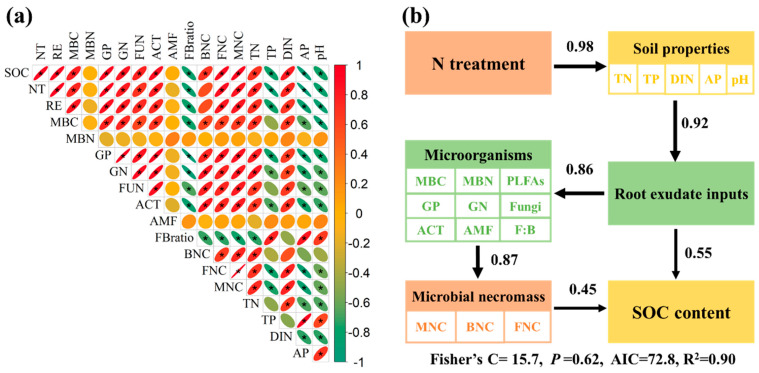
Pearson correlation matrix (**a**) illustrating the relationships among nitrogen application rate (NT), root exudate carbon inputs (RE), soil microbial community composition, microbial necromass carbon, and soil physicochemical properties. Structural equation model (**b**) depicting the direct and indirect pathways through which these variables influence SOC accumulation in paddy soil. Abbreviation: soil organic carbon (SOC), NT (nitrogen treatment), RE (root exudates), MBC (microbial biomass carbon), MBN (microbial biomass nitrogen), GP (Gram-positive bacteria), GN (Gram-negative bacteria), FUN (Fungi), ACT (actinomycetes), AMF (arbuscular mycorrhizal fungi), FB ratio (fungal-to-bacterial ratio), BNC (bacterial necromass carbon), FNC (fungal necromass carbon), TN (total nitrogen), TP (total phosphorus), DIN (dissolved inorganic nitrogen), AP (available phosphorus). Red and green indicate positive and negative correlations, respectively, with darker colors representing stronger correlations. The significance levels are noted by asterisks (*p* < 0.05).

Pearson correlation analysis indicated that SOC was positively correlated with nitrogen treatment, root exudate inputs, MBC, microbial community composition (GP, GN, fungi, and ACT), microbial necromass (BNC, FNC, and MNC), and N nutrients (TN and DIN), while negatively correlated with the F:B ratio, soil pH, and phosphorus nutrients (TP and AP; Figure 5a). Furthermore, structural equation modeling (SEM) revealed that nitrogen application altered soil physicochemical properties, which subsequently influenced root exudate inputs. Root exudates, in turn, directly affected SOC content and indirectly regulated SOC accumulation by influencing microbial communities and microbial necromass. Collectively, these parameters explained 90% of the variation in SOC content (Figure 5b).

## 4. Discussion

Understanding how N fertilization regulates SOC accumulation is essential for optimizing nutrient management and enhancing the C sequestration potential of paddy ecosystems [30,42]. Our results demonstrated that long-term N fertilization significantly increased SOC content, particularly under N200 and N300 treatments (Figure 4), suggesting that sustained N inputs promote SOC accrual in intensively managed rice systems. In addition, fine root biomass increased substantially with N application levels, indicating that N fertilization stimulated belowground biomass allocation and root system development (Table S1). Higher root biomass is generally associated with greater inputs of root residues and rhizodeposits, which serve as important C precursors for microbial growth, necromass formation, and ultimately SOC stabilization [43,44,45]. These patterns collectively suggest that rhizosphere C dynamics may represent a critical linkage between N fertilization and SOC accumulation in paddy soils [11,19,46].

Root exudates constitute a quantitatively significant and chemically diverse pathway of rhizodeposition, functioning as a critical biochemical interface that mediates plant–soil–microbe interactions in the rhizosphere [8,25]. In this study, N application significantly increased annual root exudate C flux, reflecting an N-induced shift in plant C allocation toward enhanced belowground investment (Figure 1). This response is consistent with the resource optimization hypothesis, which posits that plants increase rhizosphere C expenditure when facing stoichiometric nutrient imbalances [37,47]. Indeed, the concurrent decline in soil available P under elevated N application (Figure 4e) suggests that progressive N enrichment may have intensified relative P limitation, thereby triggering compensatory root exudation to enhance phosphatase activity and organic P mobilization [17,37,47]. The resulting increase in labile C substrate availability is known to stimulate rhizosphere microbial growth and activity (Figure 2a), potentially contributing to greater microbial biomass accumulation and SOC accrual in paddy soils [5,30].

The response of microbial communities to N fertilization provides critical mechanistic insight into how nutrient application drives SOC accumulation [11,28]. Total PLFAs increased progressively with N application rate, indicating a broad expansion of microbial biomass and metabolic activity under enhanced nutrient and substrate availability (Figure 2). Parallel increases in bacterial, actinomycete, and fungal PLFAs suggest that N application collectively stimulated the major microbial guilds responsible for organic matter decomposition and C transformation, likely reflecting the combined effects of elevated root exudate supply and improved N stoichiometry for microbial anabolism [19,27]. However, the fungal-to-bacterial ratio declined under high N application, revealing that N fertilization restructured microbial community composition (Figure 2i). This shift toward greater bacterial dominance is consistent with well-established ecological theory. Bacteria are generally characterized by higher growth rates and stronger affinity for labile C substrates compared to fungi, and thus tend to be preferentially stimulated when readily available C inputs [48,49,50].

Microbial necromass is increasingly recognized as a dominant precursor of persistent SOC, particularly in systems characterized by high microbial turnover and strong organo-mineral interactions [11,29]. Our results showed that MNC increased progressively along the N application gradient, with the highest values observed under N300 (Figure 3), suggesting that N-induced stimulation of microbial growth and turnover accelerated necromass accumulation in paddy soils [19,28,51]. The increases in both bacterial and fungal necromass indicate that multiple microbial guilds contributed to the buildup of microbially derived organic matter (Figure 3). The paddy soil environment may further amplify necromass persistence, as alternating flooded and drained conditions periodically suppress aerobic decomposition and promote the formation of reactive Fe-Al mineral phases that stabilize microbial residues through organo-mineral associations [20,32]. The concurrent increases in root-derived C inputs, microbial biomass, MNC, and SOC also suggest that microbial residue accumulation is an important pathway through which N fertilization contributes to SOC accrual [8,23].

The relationship between N fertilization and SOC accumulation should nevertheless be interpreted in the context of application rate and environmental trade-offs. Moderate N application can enhance plant productivity, belowground C inputs, microbial biomass, and necromass production, thereby promoting SOC accrual [11,52,53]. However, excessive N fertilization may induce soil acidification, nutrient imbalance, and shifts in microbial community composition that could reduce soil ecological stability [54,55]. In this study, soil pH and available P declined under high N application, indicating potential negative consequences of long-term intensive fertilization. Moreover, in paddy ecosystems, high N inputs may increase the risk of nitrous oxide emissions, particularly during drainage or drying periods, which could partially offset the climate benefits associated with SOC accumulation [56,57]. Therefore, SOC accrual alone should not be interpreted as evidence of an overall climate benefit. Instead, the C sequestration potential of N fertilization should be evaluated together with crop productivity, nutrient-use efficiency, soil health, and greenhouse gas emissions [58,59].

This study provides mechanistic evidence that N fertilization promotes SOC accumulation through coordinated increases in root exudate C inputs, microbial biomass, and microbial necromass. However, several aspects warrant further investigation. First, root exudation is dynamic and sensitive to environmental conditions and plant growth [60,61]. Future studies should increase sampling frequency or use automated systems to better capture temporal variation. Additionally, exudate composition was not characterized. Since different compounds may differentially affect SOC priming under N application, integrating metabolite profiling with ^13^C tracing would help quantify root-derived C transfer into microbial and stable SOC pools [7,62]. Second, SOC was measured only as a bulk pool, leaving C partitioning between particulate organic carbon (POC) and mineral-associated organic carbon (MAOC) unresolved. Understanding how N fertilization affects this allocation is key to assessing long-term SOC stabilization. Physical fractionation combined with biomarkers and isotope tracing would clarify the contributions of root-derived and microbial C to different SOC fractions [63,64]. Third, future studies should integrate SOC dynamics with greenhouse gas flux measurements and incorporate root exudation and microbial necromass formation into process-based models. Such integrated approaches would improve our ability to evaluate the net climate implications of N fertilization and predict SOC responses under future environmental conditions [65,66,67].

## 5. Conclusions

Enhancing SOC sequestration is central to both climate change mitigation and sustainable agricultural development. Our decade-long paddy field experiment implies that long-term N fertilization promotes long-term SOC accumulation by activating exudate-mediated microbial necromass formation pathways. Specifically, optimized N input stimulates root exudation, which fuels microbial biomass growth and turnover, ultimately facilitating the preferential stabilization of microbial necromass as persistent SOC. These findings elucidate a previously underappreciated mechanistic linkage between N fertilization and SOC persistence, providing empirical and theoretical support for optimizing N management as a practical strategy to enhance C sequestration in intensively managed paddy ecosystems.

## Data Availability

The data presented in this study are available on request from the corresponding author due to privacy concerns.

## Supplementary Materials

The following supporting information can be downloaded at: https://www.mdpi.com/article/10.3390/microorganisms14061295/s1, Table S1: Effects of N application rates on root exudate inputs.
