# Supplementary material for: Root Exudates Shape Soil Organic Carbon Stabilization by Controlling Microbial Necromass Formation Under Long-Term Nitrogen Fertilization
Source: Microorganisms. 2026 Jun 8;14(6):1295. doi: 10.3390/microorganisms14061295 (PMC13304206; doi:10.3390/microorganisms14061295)
Supplement: Supplementary file 1 [file microorganisms-14-01295-s001.zip › microorganisms-4355055-supplementary.pdf]

Supporting information

Table S1 Effects of N application rates on root exudate inputs

Treatment	Growth stages	Root exudates (μg C g h^{-1})	Root biomass (g m^{-2})	Annual C flux (g m^{-2} a^{-1})
N0	Tillering	255.9±11.7	68.0±6.2	75.8±5.1
	Booting	195.0±7.0	162.7±9.2	
	Filling	119.4±9.0	247.8±32.1	
N100	Tillering	291.7±10.3	76.0±3.4	98.5±5.5
	Booting	224.4±12.2	204.5±27.4	
	Filling	135.3±8.5	256.1±33.6	
N200	Tillering	375.4±12.6	132.5±7.9	167.7±5.8
	Booting	304.8±14.7	190.4±11.7	
	Filling	204.5±8.6	328.7±10.9	
N300	Tillering	382.3±7.0	136.4±15.5	177.4±14.3
	Booting	293.4±4.5	199.6±14.8	
	Filling	200.3±3.5	368.7±36.2	
